# Peak Serum AST Is a Better Predictor of Acute Liver Graft Injury after Liver Transplantation When Adjusted for Donor/Recipient BSA Size Mismatch (ASTi)

**DOI:** 10.1155/2014/351984

**Published:** 2014-06-09

**Authors:** Kyota Fukazawa, Seigo Nishida, Ernesto A. Pretto

**Affiliations:** ^1^Department of Anesthesiology, College of Physicians and Surgeons of Columbia University, 630 West 168th Street, New York, NY 10032-3784, USA; ^2^Division of Liver and Gastrointestinal Transplant, Department of Surgery, University of Miami Miller School of Medicine and Jackson Memorial Hospital, 1801 NW 9th Avenue, Miami, FL 33136, USA; ^3^Division of Solid Organ Transplantation, Department of Anesthesiology, Perioperative Medicine and Pain Management University of Miami Miller School of Medicine, 1611 NW 12th Avenue, D318, Miami, FL 33136, USA

## Abstract

*Background*. Despite the marked advances in the perioperative management of the liver transplant recipient, an assessment of clinically significant graft injury following preservation and reperfusion remains difficult. In this study, we hypothesized that size-adjusted AST could better approximate real AST values and consequently provide a better reflection of the extent of graft damage, with better sensitivity and specificity than current criteria. *Methods*. We reviewed data on 930 orthotopic liver transplant recipients. Size-adjusted AST (ASTi) was calculated by dividing peak AST by our previously reported index for donor-recipient size mismatch, the BSAi. The predictive value of ASTi of primary nonfunction (PNF) and graft survival was assessed by receiver operating characteristic curve, logistic regression, Kaplan-Meier survival, and Cox proportional hazard model. *Results*. Size-adjusted peak AST (ASTi) was significantly associated with subsequent occurrence of PNF and graft failure. In our study cohort, the prediction of PNF by the combination of ASTi and PT-INR had a higher sensitivity and specificity compared to current UNOS criteria. *Conclusions*. We conclude that size-adjusted AST (ASTi) is a simple, reproducible, and sensitive marker of clinically significant graft damage.

## 1. Introduction

Despite the marked advances in the perioperative management of the liver transplant recipient, an assessment of clinically significant graft injury following preservation and reperfusion remains difficult [1]. The lack of a sensitive clinical marker of acute liver injury has a profound impact on clinical practice and the success of translational research in liver transplantation. Such a clinical marker could aid the management of graft dysfunction in early identification of recipients who will require retransplantation. Currently, posttransplant peak aspartate aminotransferase (AST) is a widely accepted clinical marker for graft damage in liver transplant practice [2–6]. In fact, the United Network for Organ Sharing (UNOS) suggests relisting criteria for the recipients with primary graft dysfunction (PNF) on the basis of the post-transplant peak AST [7]. AST is an enzyme that is involved in amino acid metabolism, primarily existing in hepatocytes. During transplantation graft hepatocytes are inevitably injured, and intracellular enzymes are subsequently released into the systemic circulation of the recipient. We theorized that the recipient AST serum concentration is a function of the total amount of AST released by the graft liver diluted by the total circulating blood volume at the time of blood sampling. Therefore, the AST value could be misleading or inaccurate, depending on the size of the graft (total mass of injured hepatocytes) and the volume of blood in the recipient's circulation.

We have previously reported on the use of the ratio of donor to recipient body surface area (BSAi) to predict donor-recipient graft size mismatch [8, 9]. We showed that size mismatch has a bidirectional impact on graft survival with a progressive increase in the risk of graft failure towards both ends of the size-mismatch spectrum, small-for-size to large-for-size 5 by generalized additive model [9]. BSAi has an important value in deceased liver transplant to quickly and reliably predict size-mismatch-related comorbidities and graft outcome.

In this study, we hypothesized that serum AST when adjusted by the ratio of the donor-to-recipient body surface area (ASTi) could more accurately approximate the true mass of injured hepatocytes than the uncorrected or absolute peak AST, subsequently providing a more precise assessment of graft fate. In order to test this hypothesis, we conducted a study to investigate the sensitivity and specificity of ASTi versus absolute peak AST to predict PNF and graft survival in a large cohort of postliver transplant patients.

## 2. Methods

After institutional review board approval, primary retrospective data collection was from patient records who underwent whole orthotopic liver transplantation (LT) from January 2002 to March 2008 at our institution (Jackson Memorial Hospital, University of Miami, Leonard Miller School of Medicine, Miami, FL, USA). We were interested in the effect of size-matched peak AST (ASTi) on postoperative outcomes. Therefore, among all transplant recipients, we excluded those who underwent split or partial LT as well as those who underwent simultaneous other organ transplantation, such as liver-kidney, liver-heart, liver-intestine, or multivisceral transplantation or live donor LT. The donor risk index was calculated without height [10]. We used the previously reported index “body surface area index (BSAi)” to investigate donor-recipient size mismatch on postoperative primary graft dysfunction [8, 9]. The BSAi was calculated by the following equation:

(1) $$\begin{array}{c} \text{BSA}=\text{Weight}\;{\left(\text{kg}\right)}^{0.425}\times \text{Height}\;{\left(\text{cm}\right)}^{0.725}\times 0.007184\\ \text{BSAi}=\frac{\text{Donor}\;\text{BSA}}{\text{Recipient}\;\text{BSA}}. \end{array}$$

Size-adjusted peak AST (ASTi) was calculated by dividing peak AST by BSAi. Peak AST was defined as maximum AST within 7days after LT. 

*Statistical Analysis.* Primary nonfunction (PNF) of liver graft is the most detrimental condition after transplantation. We defined PNF as retransplantation within 7 days without vascular thrombosis. UNOS suggests the listing criteria for recipients with PNF as an AST ≥3,000 and a PT-INR ≥2.5 or acidosis pH ≤7.30 (arterial), pH ≤7.25 (venous), and/or lactate ≥4 mMol/L or anhepatic candidate within 7 days after implantation of a graft [7]. Of these criteria, the combination of AST and PT-INR was used for comparative purposes in this study. Scatter plotting was used to show the statistical association between AST and ASTi with subsequent development of PNF. The area under the receiver-operating-characteristic curve (AUC) was used to evaluate diagnostic accuracy of ASTi relative to that of conventional AST. The predictive value of current UNOS criteria for PNF (by combination of AST and PT-INR) was assessed by sensitivity and specificity in our study cohort.

The logistic regression model was used to describe the association between seven study groups differentiated by ranges of ASTi and the incidence of PNF. The patient group with ASTi <1,000 was used as a reference group. All recipient and donor variables were included in the multivariable model. To assess the independence of ASTi effect on PNF, a backward conditional elimination procedure was then undertaken using the donor and recipient demographic factors. Cox proportional-hazards models were used to examine the association between seven study groups differentiated by ranges of ASTi and graft survival; data were censored at the time of the last visit or patient death. One-year graft survival was used to assess the short- and mid-term effect of ASTi. All recipient and donor variables were included in the multivariable model. Covariates were analyzed by using a backward conditional method with the donor and recipient demographic factors. Kaplan-Meier survival analysis with generalized Wilcoxon analysis was used to assess the difference in graft survival between ASTi ≥3,500 and ASTi <3,500. SPSS statistics version 17.0 (SPSS Inc., Chicago, IL) and Intercooled STATA statistics version 11.0 (Stata Corp., College Station, TX) were used for statistical analysis and all reported *P* values are two-sided, and *P* values of less than 0.05 were considered to indicate statistical significance.

## 3. Results

A total of 930 whole LT procedures performed from January 2002 to March 2008 were reviewed in this study. 717 out of 930 consecutive whole OLT performed from January 2002 to March 2008 were included in this study. A total of 213 patients were ultimately excluded from the analysis for the following reasons: live donor LT (1 case), split LT (24 cases), auxiliary partial LT (APOLT, 6 cases), fulminant hepatic failure (14 cases), and simultaneous other organ transplants (55 cases) as well as missing data (113 cases).

*Baseline Characteristics.* The base-line characteristics of the recipients and donors of LT are summarized in Table 1. Overall average BSAi in our cohort was 0.99 ± 0.01. 

**Figure 1:**
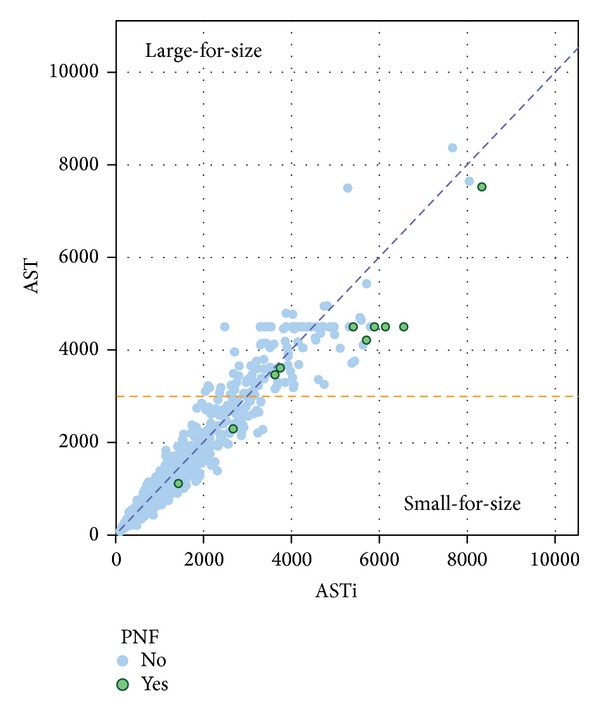
Scatter plot between AST and ASTi with or without subsequent development of PNF. Scatter plot showing the relationship between peak AST and size-adjusted AST (ASTi) for cases with or without subsequent development of PNF. Diagonal blue dot line shows 1 : 1 correlation between AST and ASTi. All the cases on the line had no size mismatch between donor and recipient. Cases above this reference line had a large-for-size graft and below the line had a small-for-size graft. All cases of PNF had small-for-size donor, and severity of graft damage may be underestimated by unadjusted AST. Orange dot line is reference line of AST 3,000 IU/L, which is currently used for diagnosis of PNF by UNOS.

*Scatter Plot between AST and ASTi with or without Subsequent Development of PNF.* Figure 1 shows a scatter plot of the relationship between peak AST and size-adjusted AST (ASTi) for cases with or without subsequent development of PNF. There were 14 cases of PNF (2.4%) in our cohort. Diagonal blue dot line shows 1 : 1 correlation between AST and ASTi. All the cases on the line had no size mismatch between donor and recipient. Cases above this reference line had a large-for-size graft and below the line a small-for-size graft. Orange dot line is the reference line for AST = 3,000 IU/L, which is the current criterion for relisting as PNF suggested by UNOS.

**Figure 2:**
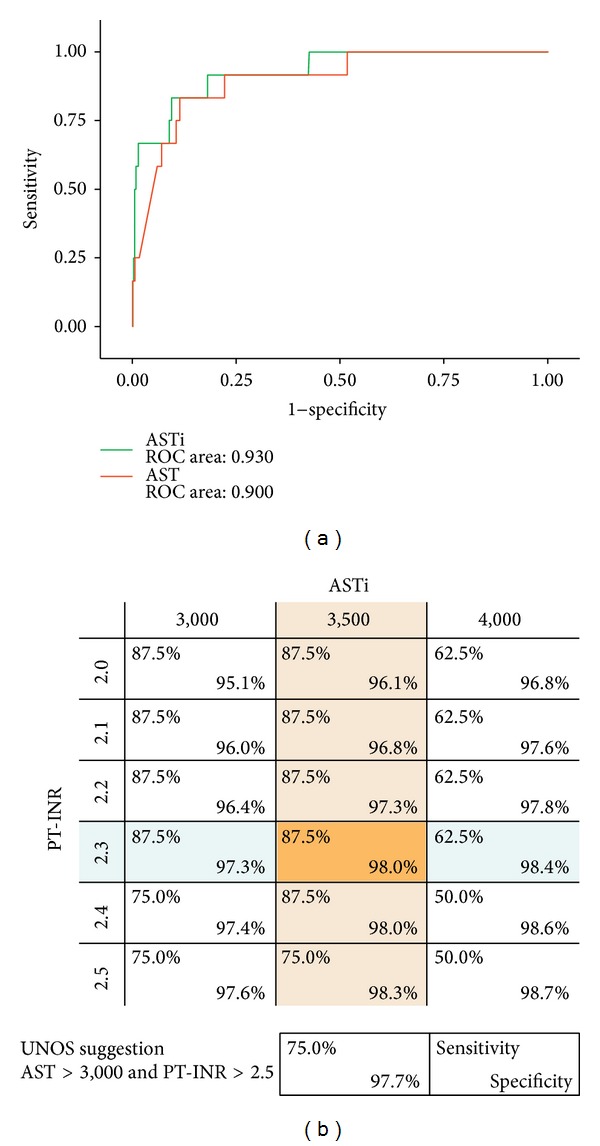
Prediction of PNF by AST and ASTi. Receiver-operating-characteristic (ROC) curves for AST and adjusted AST (ASTi) at risk for primary nonfunction of graft (PNF) show the relationship between true positive and false positive rates for a test across various threshold values used to diagnose a condition. The area under the curve (C statistic) for the ROC shown in ASTi is 0.930. The C statistic for AST in the same cohort was 0.900. ASTi is better predicting posttransplant PNF. UNOS criteria of PNF had sensitivity of 75% and specificity of 97.7% in our cohort of study population. Combination of ASTi ≥3,500 IU/L and PT-INR ≥2.3 had the best predictive value of sensitivity 87.5% and specificity of 98.0%, which is better than UNOS criteria.

*Prediction of PNF by AST and ASTi.* A receiver-operating-characteristic (ROC) curve for AST and adjusted AST (ASTi) at risk for PNF is shown in Figure 2. ROC curves show the relationship between true positive and false positive rates for a test across various threshold values used to diagnose a condition. The area under the curve (C statistic) for the ROC shown in ASTi is 0.930. The C statistic for AST in the same cohort was 0.900. ASTi is better at predicting posttransplant PNF. UNOS criteria of PNF had a sensitivity of 75% and specificity of 97.7% in our study cohort. The combination of ASTi ≥3,500 IU/L and PT-INR ≥2.3 had the best predictive value, with a sensitivity of 87.5% and a specificity of 98.0%, which was better than that obtained with UNOS criteria.

*Cases with Different Predictive Results for PNF between Conventional UNOS Criteria and ASTi Criteria.* Improvement of prediction for PNF was further assessed by a case-by-case comparison of results between conventional UNOS criteria and ASTi criteria (Table 2). Seven cases were identified as the cases which had different results between conventional UNOS criteria and new ASTi criteria. Five cases had better results with new ASTi criteria and two cases had better results with conventional UNOS criteria. Of three cases with the diagnosis of small-for-size donor, one case had a better result with ASTi due to improvement by size adjustment. Of four cases with the diagnosis of large-for-size donors, three cases had better result with ASTi for the same reason.

**Figure 3:**
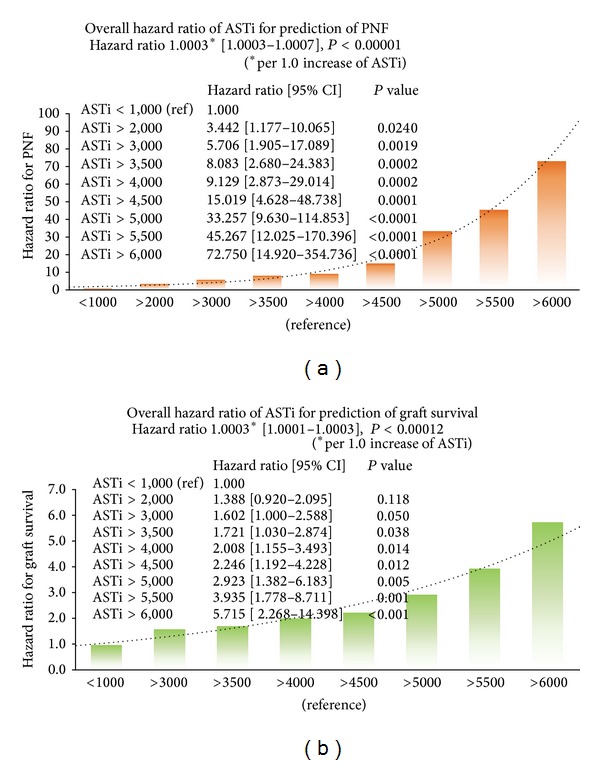
Predictive value of ASTi for primary nonfunction and one-year graft survival. (a) A logistic regression model showed that ASTi has significant impact on PNF (hazard ratio 1.0003 (95% CI: 1.0003–1.0007), *P* = 0.00001). If ASTi is greater than 2,000 IU/L, there is an exponential increase of hazard ratio for PNF along with increased ASTi. (b) A Cox proportional hazard model showed that ASTi has significant impact on graft survival (hazard ratio 1.0002 (95% CI: 1.0001–1.0003), *P* = 0.00012). If ASTi is greater than 3,500 IU/L, there is an exponential increase of hazard ratio for PNF along with increased ASTi.

*Predictive Value of ASTi for Primary Nonfunction of Graft in Logistic Regression Model.* Predictive values of different ASTi cutoffs for the primary nonfunction of the graft were analyzed with a logistic regression model (Figure 3(a)). A logistic regression model showed that ASTi has significant impact on PNF (hazard ratio 1.0003 (95% CI: 1.0003–1.0007), *P* = 0.00001). If ASTi was greater than 2,000 IU/L, there was an exponential increase of hazard risk ratio for PNF along with increased ASTi. 

*Predictive Value of ASTi for One-Year Graft Survival in the Cox Proportional Hazard Model.* Predictive value of different ASTi cutoffs for one-year graft survival was analyzed with Cox proportional hazard model (Figure 3(b)). A Cox proportional hazard model showed that ASTi has significant impact on graft survival (hazard ratio 1.0002 (95% CI: 1.0001–1.0003, *P* = 0.00012). If ASTi is greater than 3,500 IU/L, there is an exponential increase of hazard risk ratio for PNF along with increased ASTi. 

**Figure 4:**
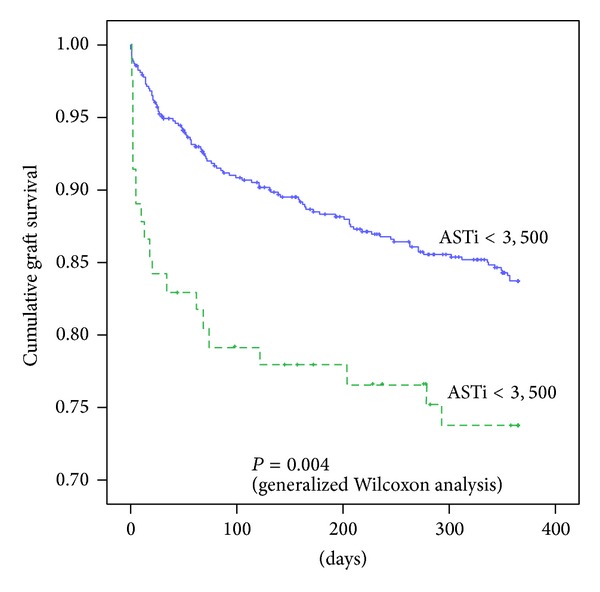
Significant reductions in one-year graft survival in patients with ASTi >3,500 IU/L compared to patients with ASTi <3,500 IU/L. Kaplan-Meier survival analysis showed the probability of primary graft outcome among patients who had a peak-adjusted AST (ASTi) of greater than 3,500 IU/L along with those who had ASTi of less than 3,500 IU/L. The probability of event-free graft survival at 1 year was 0.576 in the ASTi >3,500 IU/L group and 0.487 in the ASTi <3,500 IU/L group. There was a significant reduction in graft survival in ASTi >3,500 IU/L group.

*Comparison of One-Year Graft Survival in Patients with ASTi ≥3,500 IU/L and ASTi <3,500 IU/L.* Because ASTi had significant impact on one-year graft survival, we divided our study cohort into two groups: ASTi ≥3,500 IU/L and ASTi <3,500 IU/L. Then graft survival was compared by Kaplan-Meier survival analysis with generalized Wilcoxon analysis. Kaplan-Meier survival analysis showed the probability of the primary graft outcome among patients who had a peak-adjusted AST (ASTi) of greater than 3,500 IU/L along with those who had an ASTi of less than 3,500 IU/L (Figure 4). The graft survival at 1 year was 73.8% in the ASTi >3,500 IU/L group and 83.7% in the ASTi <3,500 IU/L group. There was a significant reduction in graft survival in ASTi >3,500 IU/L group (*P* = 0.004).

## 4. Discussion

Despite the significant advances in the management of the liver transplant (LT) recipient, the quantitative assessment of graft damage remains uncertain [1]. Acute injury and inflammation of transplanted organs during the immediate postoperative period may be linked to early organ dysfunction resulting in higher graft failure rates and rejection in the recipient [11]. Therefore, development of appropriate clinical markers to identify organ injury in the early postoperative period will have a profound impact on patient outcomes as well as on the success of clinical and translational research [11, 12]. Such clinical markers of liver damage can be of assistance in identifying the mechanisms of liver inflammation and injury and predisposing factors of donor organ injury, making it possible to implement injury-specific treatment strategies to promote organ resuscitation. In response to the transplant community's urgent need for a useful clinical marker of injury, ongoing NIH clinical trials in organ transplantation have attempted to investigate posttransplant genomic expression patterns, which may correlate with a high probability of graft dysfunction or failure [13].

Currently, in LT practice posttransplant peak AST is the most widely accepted clinical markers of graft damage. UNOS defines PNF mainly based on the posttransplant peak AST [7]. AST is an enzyme that is involved in amino acid metabolism, primarily existing in liver as well as heart, skeletal muscles, kidney, and brain. When hepatic parenchymal cells are injured, AST is released into the systemic circulation, causing an elevation of serum AST. However, the serum AST level depends on the mass of the donor liver graft (the degree and extent of injured and dying hepatocytes) and on the recipient circulatory blood volume. The working hypothesis that guided this study is that the peak AST “corrected” by the ratio of donor-to-recipient body surface area could be determined to be more accurately associated with the degree of graft injury, thus better predicting graft outcome in LT.

Indeed, the results of this study prove that the size-adjusted peak AST (ASTi) has higher sensitivity and specificity to predict PNF compared to size-unadjusted AST. Currently UNOS suggests for relisting criteria of recipients with PNF as AST ≥3,000 IU/L and either (a) INR ≥2.5 or arterial pH ≤7.30 or venous pH ≤7.25 and/or lactate ≥4mmol/L, (b) anhepatic patient within 7 days after transplantation [7]. In our study cohort, when we applied UNOS criteria, we found a relatively low sensitivity of 75.0% and specificity of 97.7%. This relatively high false positive result with conventional UNOS criteria may be due to the fact that some cases of PNF occurred in small-for-size donors as shown in Figure 1, where AST is more likely to underestimate the severity of graft damage based on the reasoning previously described. Also, we found that the severity of graft damage in large-for-size donors can be overestimated by size-unadjusted AST. In fact, one case was underestimated and three cases were overestimated by conventional criteria and this “error” was corrected by ASTi criteria as shown in Table 2. When we used size-adjusted AST (ASTi), the combination of size-adjusted ASTi and PT-INR predicted PNF more accurately, and the sensitivity increased to 87.5% with a specificity of 98.0% at cutoff of ASTi ≥3,500 IU/L and PT-INR ≥2.3. In addition, ASTi correlated well with the incidence of PNF and short-term graft survival. Increases in ASTi were significantly associated with increasing incidence of PNF. Also ASTi was highly correlated with the incidence of 1-year graft failure, if ASTi exceeded 3,500 IU/L. A more sensitive prediction of PNF with ASTi will enable clinicians to more accurately identify the recipient who requires retransplantation, perhaps, at an earlier stage in the postoperative period, resulting in better patient outcome. One-year graft survival (shown in Figure 4) showed that ASTi >3,500 IU/L group was associated with a steep decrease in graft survival in the early postoperative period compared to ASTi <3,500 IU/L group. These findings are consistent with a high predictability of graft failure. These findings suggest that in LT markers of postoperative graft injury should be interpreted with caution by taking into account graft size mismatches.

BSAi appears to be a powerful tool to detect donor-recipient size mismatch in cadaveric LT but awaits further validation. Small-for-size or large-for-size grafts not only have been attributed to decreased graft survival, increased additional graft usage (retransplantation), and increased incidence of other complications but also have made an assessment of posttransplant graft injury difficult. This study has demonstrated that size mismatch may have an even greater negative impact on graft outcome in the presence of other donor risk factors, such as prolonged cold ischemia. Furthermore, the lack of a suitable clinical measure to reliably predict size mismatch has probably hindered our ability to judge its clinical importance in cadaveric LT. BSAi provides a simple, reproducible, and sensitive way of detecting size mismatch in cadaveric LT.

We are aware that our study has some limitations. The primary limitation of this study is its retrospective nature. Second, fluid excess (ascites and edema) is universal in end-stage liver disease. Fluid excess causes weight-to-height parameters such as percentage ideal body weight, BMI, and BSA to be underestimated, and the BSA of the recipient may not accurately reflect the liver volume in these situations. These discrepancies will be carried over to the calculations of ASTi. Alternative ways to more accurately detect size disparity may be (i) measuring the donor graft weight at back table or (ii) using the recipient's dry weight instead of recipient BSA. However, we believe that ASTi is a simple, reproducible, and sensitive predictor of graft outcome associated with size mismatch. Third, adjustment of postoperative liver enzyme by BSAi requires some caution. The liver has the excellent regenerative capacity even in the face of significant loss of hepatocyte mass. This has been demonstrated after liver resection or traumatic hepatic injury [14, 15]. Once mass (volume) or functional compensation starts, BSAi may no longer accurately reflect the size mismatch between donor and recipient. In experiments with rodents, regeneration of liver mass occurred by 3 days after hepatectomy and restoration is complete by 5–7 days [16]. The human liver seems to recover the mass slower than rodents. Recent clinical observations showed that recipients of partial grafts have a rapid proliferation of liver mass, with a majority reaching a calculated standard liver volume by one month [17, 18]. Although several factors have been proposed to explain the delay in the regeneration process, such as small-for-size donor, severity of ischemic injury, immunosuppressive medications, presence of steatosis, and older age [15], for these reasons the use of BSAi-adjusted liver enzymes as more sensitive and specific markers of liver injury should be limited to the immediate postoperative period. Lastly, although ASTi seems to be a useful index to predict outcome in adult OLT, it remains unclear whether ASTi is also useful in pediatric OLT. Since SLV calculated with BSA can be applied to pediatric populations [19], ASTi should also be applied to pediatric patients and should provide useful information for preoperative estimation of liver grafts regardless of age. However, predicting outcome by BSAi for pediatric population warrants further investigation.

We conclude that the ASTi appears to be a sensitive predictor of postoperative liver graft injury in the early postoperative period. Despite some shortcomings, we believe our analysis shows that the ASTi may provide significantly better prediction of size mismatch-related graft dysfunction in LT. Finally, we believe that ASTi should be considered in the early identification of graft dysfunction or PNF and should be validated in a prospective study to see whether it accurately and consistently predicts PNF or graft function and reduces posttransplant mortality.

## Figures and Tables

**Table 1:** Recipient and donor variables.

Variables	Mean ± SEM	(*N* ^†^)
Recipient		
Age	52.8 ± 0.42	(717)
Percentages of male (%)	31.6%	(717)
Standard liver volume (mL)	1086.5 ± 7.1	(717)
Cause of end-stage liver disease		(717)
HCC	27.8%	
HBV	8.4%	
HCV	51.3%	
Alcoholic	20.2%	
PBC/PSC	1.0%	
AIH	3.2%	
Cryptogenic	1.4%	
MELD	21.6 ± 0.3	(717)
PT (sec)	18.3 ± 0.2	(709)
Fibrinogen (mg/dL)	194.4 ± 5.3	(445)
Creatinine (mg/dL)	1.3 ± 0.0	(710)
Total bilirubin (mg/dL)	7.4 ± 0.4	(704)
Sodium (mEq/L)	136.7 ± 0.2	(710)
Platelet (×10^3^ cells/mL)	99.1 ± 2.9	(711)
Donor		
Age	41.1 ± 0.7	(717)
Percentages of male (%)	40.0%	(717)
Standard liver volume (mL)	1053.1 ± 6.9	(717)
Cause of death		(717)
Anoxia (%)	9.5%	
Cerebrovascular accident (%)	7.1%	
Cold ischemia time (min)	413.9 ± 3.9	(713)
Warm ischemia time (min)	39.6 ± 0.4	(711)
Donor risk index^¶^	1.78 ± 0.01	(670)
BSAi (donor/recipient)	0.99 ± 0.01	(717)

Data are means ± SEM or percentages. ^†^Number of patients with data available. ^¶^Donor risk index without height [10] AIH: autoimmune hepatitis; MELD: BSAi: body surface area index [8]; HBV: hepatitis B virus; HCC: hepatocellular carcinoma; HCV: hepatitis C virus; models for end-stage liver disease; PBC: primary biliary cirrhosis; PSC: primary sclerosing cholangitis; PT: prothrombin time.

**Table 2:** Analyses of cases with different predictive results for PNF by conventional UNOS criteria and ASTi criteria.

				Conventional criteria						ASTi criteria	
	PNF	AST	PT-INR	AST 3,000	Results		BSAi	ASTi	PT-INR	ASTi 3,500	Results
	(Yes: 1, no: 0)	(mg/dL)	(—)	PT-INR 2.5			(—)	(mg/dL)	(—)	PT-INR 2.3	
Case 1	0	3256	2.3*	0	**TN**	Small-for-size	0.69	4745	2.3	1	FP
Case 2	1	4500	2.3	0	FN	Small-for-size	0.69	6556	2.3*	1	TP
Case 3	0	3233	3.9	1	FP	Small-for-size	0.99	3249*	3.9	0	**TN**
Case 4	0	4500	2.4*	0	**TN**	Large-for-size	1.02	4430	2.4	1	FP
Case 5	0	3308	3.1	1	FP	Large-for-size	1.05	3137*	3.1	0	**TN**
Case 6	0	3469	2.8	1	FP	Large-for-size	1.12	3105*	2.8	0	**TN**
Case 7	0	3760	5.0	1	FP	Large-for-size	1.17	3207*	5.0	0	**TN**

*Results for prediction of PNF by two different prediction criteria*. Bold font shows the favorable results compared to results of the other criteria. *In PT-INR or AST, ASTi represent the factors, which were responsible for improvement in results. First three cases had small-for-size donors and last four cases had large-for-size donors. AST: aspartate aminotransferase, BSAi: body surface area index, FN: false negative, FP: false positive, PNF: primary nonfunction of graft, PT-INR: international normalized ratio of prothrombin time, TN: true negative, and TP: true positive.
